# Characterizing environmental geographic inequalities using an integrated exposure assessment

**DOI:** 10.1186/s12940-021-00736-9

**Published:** 2021-05-12

**Authors:** Julien CAUDEVILLE, Corentin REGRAIN, Frederic TOGNET, Roseline BONNARD, Mohammed GUEDDA, Celine BROCHOT, Maxime BEAUCHAMP, Laurent LETINOIS, Laure MALHERBE, Fabrice MARLIERE, Francois LESTREMAU, Karen CHARDON, Veronique BACH, Florence Anna ZEMAN

**Affiliations:** 1 Institut National de l’Environnement Industriel et des Risques (INERIS), Parc ALATA BP2, 60550 Verneuil en Halatte, France; 2 PériTox, UMR_I 01, CURS, Université de Picardie Jules Verne, Chemin du Thil, 80025 Amiens, France; 3 LAMFA, UMR CNRS 7352, Université de Picardie Jules Verne, 33 rue Saint-Leu, 80039 Amiens, France

**Keywords:** Inequities, Spatial, Exposure, Modeling, Integrated

## Abstract

**Background:**

At a regional or continental scale, the characterization of environmental health inequities (EHI) expresses the idea that populations are not equal in the face of pollution. It implies an analysis be conducted in order to identify and manage the areas at risk of overexposure where an increasing risk to human health is suspected. The development of methods is a prerequisite for implementing public health activities aimed at protecting populations.

**Methods:**

This paper presents the methodological framework developed by INERIS (French National Institute for Industrial Environment and Risks) to identify a common framework for a structured and operationalized assessment of human exposure. An integrated exposure assessment approach has been developed to integrate the multiplicity of exposure pathways from various sources, through a series of models enabling the final exposure of a population to be defined.

**Results:**

Measured data from environmental networks reflecting the actual contamination of the environment are used to gauge the population’s exposure. Sophisticated methods of spatial analysis are applied to include additional information and take benefit of spatial and inter-variable correlation to improve data representativeness and characterize the associated uncertainty. Integrated approaches bring together all the information available for assessing the source-to-human-dose continuum using a Geographic Information System, multimedia exposure and toxicokinetic model.

**Discussion:**

One of the objectives of the integrated approach was to demonstrate the feasibility of building complex realistic exposure scenarios satisfying the needs of stakeholders and the accuracy of the modelling predictions at a fine spatial-temporal resolution. A case study is presented to provide a specific application of the proposed framework and how the results could be used to identify an overexposed population.

**Conclusion:**

This framework could be used for many purposes, such as mapping EHI, identifying vulnerable populations and providing determinants of exposure to manage and plan remedial actions and to assess the spatial relationships between health and the environment to identify factors that influence the variability of disease patterns.

**Supplementary Information:**

The online version contains supplementary material available at 10.1186/s12940-021-00736-9.

## Introduction

The World Health Organization in a recent report (2012) [1] has identified environmental inequities as a priority issue that needs to be addressed by the national governments of Europe. Reducing health inequities means identifying and characterizing exposure in order to interpret how they accumulate across a territory and to prioritize interventions. As the health status of a population is the result of complex interactions between several social, territorial and environmental factors, all the related information needs to be studied in order to assess it. At a regional or continental scale, the characterization of environmental health inequities (EHI) expresses the idea that populations are not equal in the face of pollution. It implies an analysis must be done in order to identify and manage areas at risk of overexposure where increasing risk to human health is suspected. The development of methods is a prerequisite for implementing public health activities aimed at protecting populations. Constructing tools to guide public action in order to reduce the EHI requires evaluating phenomena that are not always simple to comprehend and making the available information reliable and representative, which usually demands statistical processing [2].

In France, after more than 10 years of actions aimed at preventing environmental health risks, the third national plan for health and environment (PNSE 3, 2015–2019) proposes a new EHI approach that is not only more robust and connected to the territories but also integrates the scientific concept of exposome. The recently emerged term of exposome [3] is used to describe these complex exposures, considering all sources, routes, and - when possible - the interactions of stressors, that are likely to contribute to a health alteration in individuals. The external contribution to the human exposome is determined by environmental exposure, also termed the eco-exposome [4] and includes exposure from air, water, soil and food exposure media. A coherent conceptual framework for exposure assessment is needed to tackle EHI, one that permits an estimation of the magnitude, frequency and duration of exposure to chemicals, along with the number and characteristics of the population exposed.

Quantitative exposure assessment for environmental inequity characterization poses specific questions that need to be addressed:

- Identification of contamination(s) source(s);

- Characterization of exposure mechanisms (pathways and relevant routes);

- Prioritization of vulnerable populations or specific susceptible groups (e.g. infants);

The contamination process is extremely complex and varies through space and time, with localized multiple sources at a larger scale. At a regional scale, to better evaluate exposure to large chemical emissions, fate and transport models could provide both an ability to account for the pertinent spatial variability (e.g., around emissions sources or highly populated areas) and temporal variability during a specific time of contamination [5].

Exposure assessment to identify and characterize a territorialized EHI depends on the availability of data. Exposure assessment is generally complex due to a lack of data and the inherent natural variability in exposure levels, leading to uncertainty in the estimates [6]. The temporal support also differs between the available data (punctual measurements, annual averages, etc.) which also requires additional treatment. Furthermore, they often lack a common spatial support, therefore preliminary spatial analysis is required in order to homogenize them or increase their resolution. The available databases are often assembled for diverse objectives, and often re-processed using statistical methods. Spatialization and crossing these data pose several methodological difficulties and can introduce uncertainties in the cartography process carried out. For this reason, different methods and techniques are employed to specifically treat environmental databases in order to take benefit from all the available information and reduce the uncertainties.

A global overview of the limited progress achieved in the field so far has been established and reported in Table 1, based on articles retrieved from the available scientific literature regarding some aspects of the global methodology. Some more background consideration is reconsidered in the different next sections.

**Table 1:** Bibliography related to exposure modeling for a multilevel approach

References	Type of assessment	Main input data	Model	Major outcomes / breakthroughs
**Bulle et al, 2019** **[**5**]**	Life cycle impact assessment	Emission and exposure data	IMPACT World+	Novel framework that includes recent methodological advances in multiple impact categories in a consistent way by implementing the same modeling structure of fate, exposure, exposure response, and severity across ecosystem quality and human health-related impact categories.
**Ciffroy et al, 2015** **[**6**]**	Integrated Risk Assessment	Emission and exposure data	MERLIN-Expo: fate and exposure model, non-spatial model	Key points for integration across the human and environmental disciplines is the move from environmental fate and exposure estimations to the internal dose in the exposure assessment
**Nieuwenhuijsen et al, 2019** **[**7**]**	Environmental epidemiology; exposure-wide association study	Built environment, air pollution, road traffic noise, meteorology, natural space, and road traffic	Proximity models, interpolation models, Land Use Regression models, dispersion models	First large urban exposome study of birth weight that tests many environmental urban exposures. It confirmed previously reported associations for green space exposure and generated new hypotheses for a number of built-environment exposures.
**Vrijheid et al, 2020** **[**8**]**	Environmental epidemiology; exposure-wide association study	Indoor and outdoor air pollutants, built environment, green spaces, tobacco smoking, and biomarkers of chemical pollutants	Proximity models, interpolation models, Land Use Regression models, dispersion models	First comprehensive and systematic analysis of many suspected environmental obesogens strengthens evidence for an association of smoking, air pollution exposure, and characteristics of the built environment with childhood obesity risk.
**Juarez et al, 2014** **[**9**]**	Spatio-temporal and multilevel approach for examining exogenous and endogenous source-exposure-disease relationships	Natural, built, social and policy environment variables	Spatial and multi-level statistic approach	Retrospective and prospective systems theory modeling and methods, including advanced and complex multi-level, spatial, Bayesian, and high throughput mathematical designs. Use of data-driven, graph theory/combinatorial techniques and analytics from computational biology to identify relationships among the myriad of environmental exposure and population health data points.
**Teeguarden et al, 2016** **[**10**]**	Aggregate exposure assessments	Emission, environmental concentration, population behavior and physiology	Aggregate Exposure Pathway	Development of the Aggregate Exposure Pathway concept as the organizational framework for exposure science, builds on the long history of aggregate exposure assessments as a key feature of the field and recent technological advances in computational exposure modeling and informatics.
**Bravo et al, 2012** **[**11**]**	Data sampling and data reprensentativeness	Monitoring data, emission and meteorological data	Community Multi-Scale Air Quality (CMAQ) modeling system	Spatial and temporal resolution improvement and uncertainty reduction
**Malherbe et al, 2002** **[**12**]**	Data sampling and data reprensentativeness	Topsoil concentration data	Statistical (probabilistic) vs. non-statistical (directed) approaches	Procedure that could be followed to design a soil sampling strategy for human health risk assessment
**Caudeville et al, 2012** **[**13**]**	Spatial human exposure	Topsoil concentration data	Geostatistic and Modul’ERS model	Complex geostatical method used for human exposure assessment
**Chakraborty et al, 2011** **[**14**]**	Environmental justice and health risk disparities	Air concentration data, ethnicities, cancer rate	Simultaneous autoregressive (SAR) models	Spatial regression models for assessing environmental justice and health risk disparities
**Goovaerts, 2001** **[**15**]**	Spatial environmental contamination	Topsoil and parental material data	Several kriging models	Modelling of uncertainty for single continuous soil attributes. The issue of assessing the goodness of such models has rarely been addressed and criteria similar to the ones introduced here could be developed.
**Jerrett et al, 2005** **[**16**]**	Spatial environmental contamination	Emission, topology, meteorological, air concentation	Proximity models, interpolation models, Land Use Regression models, dispersion models	Review of the current state of knowledge for intraurban air pollution exposure assessment.
**Cattle et al, 2002** **[**17**]**	Spatial environmental contamination	Topsoil concentration data	Kriging model	Comparison of different inteprolation methods applied for air pollution
**Kanevski et al, 2009** **[**18**]**	Spatial environmental contamination	Spatial environmental data	Machine learning models	Application of machine learning methods for solving the problems of spatial dimension. Most machine learning literatures address on algorithms and models for solving non-spatial problems.
**Van de Kassteele et al, 2009** **[**19**]**	Spatial environmental contamination	Emission, topology, meteorological, air concentation	External drift kriging method	Combination of observations and a deterministic dispersion modeldescription to propose a model-based geostatistical interpolation procedure.
**Breiman, 2001** **[**20**]**	Spatial environmental contamination	14 variables about physicochemical soil properties	Hybrid regression-kriging fitted using Random Forest models	Application of machine learning methods for solving the problems of spatial dimension on environmental thematic
**Ioannidou et al, 2018** **[**21**]**	Integrated spatial human exposure	Water, air, soil, food, behavorial data	PLAINE and Modul’ERS	Proposition of an aggregated exposure assessment approach based on on modeling and monitoring network at a national scale. Adapted method for each environmental compartment are adapted for existing monitoring networks
**Guerreiro et al, 2016** **[**22**]**	Health impact	Emission, topology, meteorological, air concentation	Chimere and kriging model	Combining observations and chemical transport models through the use of spatial interpolation methods at a continental scale
**Ratola et Jiménez-Guerrero, 2015** **[**23**]**	Spatial environmental contamination	Emission, topology, meteorological, air concentation	Chimere and vegetation transfer model	Combining venegetation concentration observations and chemical transport models through the use of transfer model
**Pennington et al, 2005** **[**24**]**	Spatial human exposure	Emission, topology, meteorological, air concentation	IMPACT Western Europe	The model facilitates estimation of concentration profiles of dispersed contaminants and human intake at the population level. The results are presented in the form of intake fractions, the fraction of an emission that will be taken in by the entire population.
**Gerlowski et Jain, 1983** **[**25**]**	Toxicokinetic modeling and internal exposure	Physiological and exposure data	Toxicokinetic model	First review of physiologically based pharmacokinetics to increase the use of this modeling technique.
**Quindroit et al, 2019** **[**26**]**	Toxicokinetic modeling and internal exposure	Physiologicaln ingestion, inhlation and dermal exposure data	Toxicokinetic model	Global model for pyrethroids in humans using in vivo, in vitro and in silico data.

This paper presents the methodological framework developed by INERIS to identify a common framework for conceptualizing and operationalizing environmental exposures as an important step towards articulating a science of EHI.

In order to build a calculation infrastructure able to characterize the eco-exposome at the territorial level, it was necessary to solve several methodological issues: (1) Define an integrated exposure assessment framework that first requires different scientific limitations to be overcome, such as the linkage of the global source-effect chain, (2) provide statistical methods and numerical tools that would allow spatial and temporal data from existing environmental and populational databases to be processed, (3) link, adapt or develop transport and transfer models. Finally, a brief description of a key case study that was investigated is proposed in order to illustrate the integrated approach and the kind of assessments that could be performed.

## The integrated exposure assessment framework

Projects funded by the European Union under EU Framework Programme FP7 and EU Horizon 2020 related their research to existing infrastructures and data available in different European cohorts with the aim of comparing health outcomes and exposure information [7]. Moreover, they all invested dedicated specific efforts to build an integrated exposure assessment framework [8, 27–29].

**Fig. 1:**
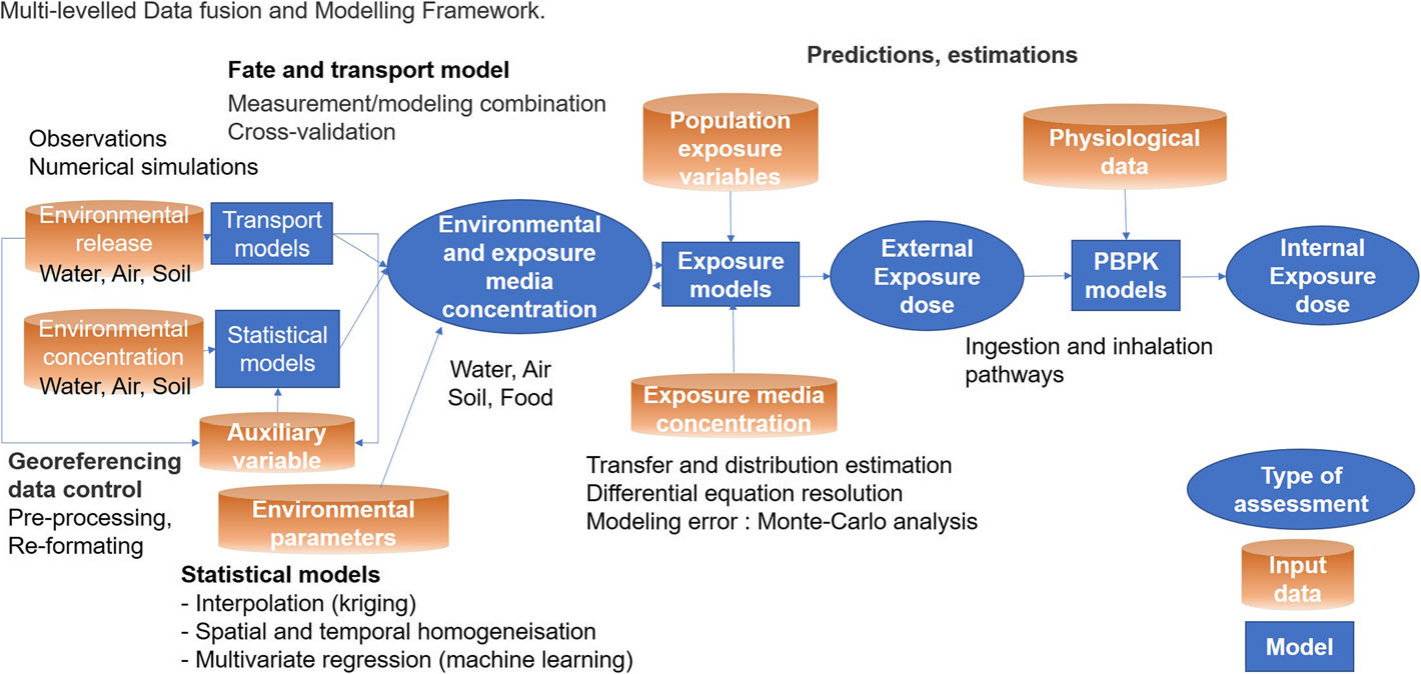
Example of a modeling framework to characterize an integrated EHI

The characterization of a territorialized exposome implies the development of dynamic, multidimensional, longitudinal approaches, and information systems that require the adoption of transdisciplinary methods of data analysis. To respond to the general objective, it is necessary to integrate and combine various levels of data from different environmental compartments and exposure media. Data and information emerging from an expanding field of exposure science can be integrated into the exposome conceptual framework. This provides the necessary linkages between source and internal exposure and helps to identify and compare relationships between different levels at critical life stages, personal health outcomes, and health disparities at a population level across space, and time [9]. This framework could be a layered structure that describes the elements of exposure pathways (Fig. 1), the relationship between those elements, and how data describing the elements is stored and used for selected outputs, such as exposure assessment, exposure prediction, epidemiology or public health decision making [10]. Refined aggregate exposure assessment is data-intensive, requiring detailed information at every step of the source-to-dose pathway. Integrated exposure assessment requires 1) methodologies to allow the aggregate exposure to be calculated systematically and 2) computational research tools to estimate the exposure from the different contributing sources.

In the context of mapping the environmental inequities enabling the identification of vulnerable individuals and communities at risk in order to target public health interventions, additional requirements are needed in the exposure assessment processes compared with classical risk assessment methodology.

The environmental inequities operate at different scales (global, regional, local) and could not be captured by studying a single medium, but by the integration of varied contamination pathways: air, water, soil and food. The design study should be able to:

- integrate the processes that take place at the interface between the environmental contaminants of interest and the organisms,

- characterize the principal exposure pathways,

- define realistic scenarios that integrate past and present sources,

- describe the phenomena at a fine temporal and spatial resolution.

Based on the needs described above, the research objectives are to bring together all the available information within a coherent methodological framework for assessing the source-to-dose continuum covering an extensive chemical space. An integrated exposure assessment approach has been developed, one that is able to integrate the multiplicity of exposure pathways from various sources, through a series of models leading up to internal exposure. The main objective of our projects, i.e. testing the feasibility of the methodology, has been achieved. Our framework allows for several matters of interest:

- identifying areas of potential overexposure by analyzing the variations of the indicators in space,

- analyzing sources and environmental components potentially associated with overexposure,

- explaining the variability of exposure inequities for pollutants and study areas,

- estimating internal exposure and linking it with human biomonitoring data.

This approach involved implementing different models, namely atmospheric dispersion modeling, spatial analysis for processing environmental and population data, a multimedia exposure model and a physiologically-based pharmacokinetic (PBPK) model. The models have been adapted and coupled to allow the integration of the output data from an upstream model as input data for the downstream model. The coupling also allows the information on the sources of contamination, the quality of environmental media and the resident populations to be integrated on the same analysis medium, namely the reference grid. A Geographic Information System (GIS) thus provides the opportunity to cross the estimated exposure with biological impregnation data to provide interpretive elements of the environmental determinants of exposure. The coupling of numerical and statistical models has established a scientific and technical basis for integrating, data processing and assessing the transfer of contaminants from the environment to populations. In that way, it is possible to integrate all available data, despite their heterogeneity, in a common spatial support. The reference grid selected, allows local variations to be reflected as well as the integration of environmental monitoring databases in France. The GIS modeling platform enables the coupling and interoperability of all spatial data via the reference grid on which the input data and the variables of interest are discretized after processing.

## In search of data and representativeness

### Integrated data from existing environmental health monitoring programs

#### Data used

Many different approaches can be used for quantifying environmental exposures: direct methods (measuring, monitoring or biomonitoring) or indirect methods, involving exposure estimations from measurements and existing data, such as environmental monitoring, questionnaires and exposure models. The quality and usability of all environmental data should be assessed before employing them in the health or risk assessment processes, as many factors can bias environmental sampling results [30]. Ideally, direct measures of exposure (e.g., biomarkers or personal monitoring data) for all key stressors related to health effects, throughout the critical time-period of exposure, and in the population of interest, would be necessary [31]. However, exclusive use of biomarker data in exposure assessment to characterize the EHI is currently not practicable when considering a large number of diverse chemicals due to analytical and resource limitations [32] specifically when the assessment should cover a large territory and give fine resolution. Environmental quality data are often available at a fine administrative or resolution level and enable environmental indicators to be built on a regional or national scale. The processing of variables for the identification and characterization of environmental inequities depends on the reuse of this type of data, which are very diverse by nature regarding their initial intended objectives. Determining how representative those measured levels of contamination are of other locations or time frames is not always a simple task [33].

Health and environmental databases have been developed for several years. They evolve and are in full expansion. Actions to identify and monitor the quality of the environment for soils, water and air are conducted by different agencies, institutes or observatories. The production of this type of data and advances in computer technology allow their reuse in conceptual frameworks and with objectives different from those that prevailed during their implementation. The emergence of quality data and their integration into GIS make it possible to conduct territorial analyses. These environmental data reflect the actual contamination of the environment and therefore the populations’ global exposure. The indicators based on these data allow a characterization of the population’s exposure and its evolution regarding the implementation of public prevention policies. In the context of reusing this type of data for the purpose of expology, a database must be set up in which the variables are associated with the modes of exposure (concentrations in the environmental and exposure media are present, eating behavior, space-time budget, etc.).

#### Data preprocessing

The processing operations to be performed usually consist of the following basic steps:

- the identification of data sources allowing the construction of different variables,

- the acquisition of these data in view of the access modalities, the financial, legal or human aspects,

- an analysis of the quality and representativeness of the databases regarding the study’s objective (choice of a database, validity and representativeness of the data) sometimes involving an approximation or the application of simplifying assumptions,

- the preprocessing of databases: cleaning the databases, rebuilding missing data,

- the construction of ad-hoc data where the appropriate data sources are not available or exhaustive in relation to the study’s objectives,

- data transformation (homogenization, aggregation or disaggregation of data).

The estimation of exposure requires knowledge of the concentrations of the environmental components to which an individual or a population is exposed. These concentrations can be measured or modeled. A wide range of data might potentially be mobilized for an integrated assessment. The database selection or study design definition should be guided to achieve the best compromise between data representativeness and method robustness, consistent with the objectives of the study.

#### Environmental monitoring networks

Characteristics of air pollution (e.g., chemical components, particle properties) vary spatially [34] and may differ between areas near and far from the monitors [11]. Automated monitoring networks operate in Europe providing detailed air quality information on a regular basis. The soil routes of exposure to humans are inhalation of dust and vapor coming from soil contaminants, ingestion of contaminated soil particles (mainly for children) or contaminated food, and dermal absorption through the skin. Once a site is considered as contaminated, it is necessary to provide enough accurate data to minimize a lack of statistical representativeness and increase the spatial quantification. The time spent for evaluating the presence and extent of contamination can be reduced by an adequate sampling plan [12] which can, at the same time, reduce the project costs [35]. A soil monitoring system could be a source of comparable and objective data on the current state and evolution of soils. The database of the soil monitoring system allows the creation and maintenance of data for each of the monitoring sites of agricultural land as well as the preparation of data for further processing through specialized programs [36]. Position information provides a link to the GIS, and thus opens the possibilities for further spatial analysis, the identification of risk areas and their assessment. For example, in France, soil pollutant stocks and properties and most explanatory variables were derived from the French National Soil Monitoring Network (Réseau de Mesures de la Qualité des Sols or RMQS). The RMQS surveys soils and their properties on a regular 16 km grid across the French mainland territory (around 2200 sites covering 550,000 km^2^) [37].

The Drinking Water Directive (80/778/EEC), and its successor (98/83/EC, which comes into force in 2003), aims to ensure that water intended for human consumption is safe. In addition to microbiological and physicochemical parameters, a number of toxic substances such as pesticides, polyaromatic hydrocarbons, cyanide compounds, and heavy metals are to be monitored. This is because the raw supply may be contaminated, for example, with pesticides from agricultural land which have leached into groundwater or from contamination within the distribution system, such as lead from piping. In France, 300,000 samples are tested each year. Indeed, tap water is one of the most strictly controlled foodstuffs. Each year, the health agencies carry out close on 12.3 million tests covering all of the country’s public water and wastewater services (both publicly and privately managed). In 2013, more than 8.1 million tests were carried out on services managed by private water companies.

Work has been carried out by INERIS to identify environmental and spatialized databases for the purpose of characterizing exposures by associating the main producers and data managers identified [38, 39]. It allows elements for the specification of environmental health platforms to be proposed and to improve the integration of data in the framework of building an environmental health tracking information system. However, spatial data used to characterize environmental exposures have not always been initially collected and collated to meet these objectives, resulting in use bias. Measuring frequencies or spatial densities of sampling are not always sufficient. The selection of a treatment method depends on the problem to be solved and the quality of the data available.

### Statistical approaches to link and optimize data representativeness

#### Spatial data properties

The data available in a region of interest characterize levels of contamination at very specific locations, over a given spatial support (i.e. the support on which the data is measured such as point, surface or volume), and for a very specific time frames. In order to construct exposure maps from spatialized databases in the context of evaluating environmental inequities, it is necessary to develop methods for processing and harmonizing the available data, with respect to their specificities (missing values, limited number of observations, etc.) at the same resolution and support.

In the mathematical field of numerical analysis, interpolation is a method of constructing new data points within the range of a discrete set of known data points. Recently, the increasing availability of spatial and spatiotemporal data has pushed the development of many spatial interpolation methods, including geostatistics [13]. Spatial interpolation includes any of the formal techniques that study phenomena using their topological, geometric, or geographic properties. Spatial dependence is the co-variation of properties in a geographical space: Features at nearby locations seem to be correlated. The fundamental principle is Tobler’s first law of geography: if the interrelation between entities increases with proximity in the real world, representation in geographical space and evaluation using spatial analysis techniques are appropriate [14]. These interactions are all stronger as the locations concerned are closer. In statistics, spatial autocorrelation measures the correlation of a georeferenced variable with itself. It makes it possible to measure the degree of similarity between neighboring observations. This spatial dependence implies the infringement of the assumptions made in the classical statistical techniques which suppose independence between the observations. Spatial dependence should also be considered as a source of information. The analysis of spatial data structures through geostatistical tools (variogram, autocorrelation analysis) is often employed to characterize the different scales of local, regional and global variability of the phenomena studied [40].

Several more sophisticated methods of spatial analysis can be applied to include additional information and take benefit of spatial and inter-variable correlation to improve data representativeness and characterize the associated uncertainty [15].

#### Spatial statistics

For air, several methods for estimating exposure to air pollutants exist, including monitor-based approaches such as proximity-based assessments and statistical interpolation, as well as land-use regression and air quality modeling [16]. Using data from existing monitoring networks remains popular, due to cost considerations, data availability, and population coverage. Such statistical methods are aimed at using multiple types of information to inform exposure estimates and allow an estimate of exposure to be made in areas far from monitors. In addition to fused data, several other approaches have been developed to estimate individual- and population-level exposures, including various interpolation methods, land use regression (LUR) models, aerosol measurements obtained from satellites, and source- and traffic-proximity analysis [11]. Stochastic methods such as kriging are preferred [17]. An issue commonly reported is the availability of data. Some databases include some limitation (as a limited number of observations for instance) and therefore it is not possible to assess the population’s exposure adequately. External drift kriging is then widely used in air and soil quality modeling, in order to combine different kinds of information to include secondary information in the model.

Machine learning uses algorithms and statistical methods to “learn” information directly from data without relying on a predetermined equation as a model. The algorithms adaptively improve their performance as the number of samples available for learning increases. Machine learning makes it possible, for example, to build a metamodel from a dataset of deterministic model outputs. The fundamental concepts of machine learning and its usages in spatially distributed data are given in Kanevskij et al. [18].

#### *The PLAINE platform:* the case of spatial exposure of Benzo [a] Pyrene in France

A GIS-based modeling platform developed by INERIS for quantifying human exposure to chemical substances (PLAINE: environmental inequities analysis platform [41]) aims to spatialize an environmental indicator related to human health using risk assessment methods and mapping environmental disparities at a fine resolution. The main aim of the PLAINE Project, developed in France, is to develop a platform of environmental and health data. This platform is developed for the systematic collection, integration, and analysis of data on emission sources, environmental contamination, exposure to environmental hazards, and population and health. Ad-hoc methodologies are used to align the available data to the same pixels. Spatial analysis and statistical methods are employed to process (georeferencing, data controlling, pre-processing, re-formatting) and assemble the databases for the purpose of the study, using R and QGIS. For example, atmospheric concentration data were collected in France in the context of regulatory surveillance for two years (2010 and 2011). An estimation of concentrations over France by the classical interpolation method could lead to a misrepresentation of the spatial distribution due to the limited number of observations. To address this issue, auxiliary variables, in the context of external drift kriging [19], were employed. The best auxiliary variable to define linear drifts was found to be the one that includes atmospheric emissions as well as population and altitude. Measurements of PAH topsoil concentrations are available through the French Soil Monitoring Network. Qualitative data on the polluted sites localization are integrated by processing distance-to-polluted soil proxy. These, along with 14 variables about physicochemical soil properties, were combined in a hybrid regression-kriging and fitted using Random Forest [20] models, and were shown to outperform the traditionally used linear regression. Due to its hydrophobic nature, B [a] P is found in water in small concentrations; therefore, the exact measurement cannot always be reported. The observations under the detection limit rate are quite high, which requires careful handling. A complex multiple imputation method was developed in order to extract the maximum information from the available measurements without introducing too much bias in the results. This makes it possible to take advantage of the temporal aspect and correlations between the substance of interest and other PAH substances. The spatial estimation of water concentrations was carried out by taking into account the multi-annual data and the network water distribution complexity using a bootstrap based expectation-maximization algorithm. The above methods permitted the construction of a representative spatial database in a 9 km^2^ grid of reference for the whole of France (550,000 km^2^) used to perform the integrated exposure assessment [21].

## Fate and transport models

### Outdoor air dispersion modeling

Atmospheric chemistry and dispersion modelling have experienced important improvements in the last two decades. Nowadays, a large variety of modelling systems and options exist, from simpler to more complex ones, covering global or regional to urban and street level scales.

Air quality models simulate the fluxes in atmospheric concentrations of air pollutants and their deposition onto the Earth’s surface by solving the transport equations that represent the emissions, advection, diffusion, transformations and removal of those air pollutants and the associated chemical species.

Contemporary air quality models can be grouped into two major categories:

- models that calculate the concentrations of air pollutants near a source (source-specific models). The Gaussian models simulate the atmospheric dispersion of non-reactive pollutants near the source (steady-state approach). Lagrangian models are also source-specific models, which treat atmospheric dispersion of reactive substances as a source-specific process;
- Eulerian models that calculate concentrations of reactive air pollutants over large areas ranging from an urban area, to a region, a continent and the globe (grid-based models).

Inputs to air quality models include the emission rates of primary air pollutants and precursors of secondary air pollutants, meteorology (three-dimensional fields of winds, turbulence, temperature, pressure, boundary layer height, relative humidity, clouds and solar radiation, etc.), and boundary conditions (baseline or background conditions). For grid-based models, an emission model is used to translate an emission inventory into a spatially distributed and temporally resolved grid structure.

As an example, INERIS used BaP as a tracer of the carcinogenic risk associated with PAH. This has been the subject of several recent studies using the CHIMERE model at the European scale [21, 23]. The population exposure estimate shows that 20% of the European population is exposed to BaP background ambient concentrations above the EU target value and only 7% live in areas with concentrations under the estimated acceptable risk level of 0.12 ng.m^− 3^. Heavy metals have also been addressed using the CHIMERE model [42], modelling Pb, Cd, As, Ni, Cu, Zn, Cr and Se air background concentrations in Europe. Evaluation of the model’s performance in order to reveal its ability to reproduce observed levels shows that more recent annual totals, information on snap activities for each metal, higher spatial resolution and a better knowledge of the temporal emission behavior are necessary to adequately model these air pollutants.

### Multimedia exposure models

Spatially resolved multimedia fate and multi pathway exposure models facilitate the prediction of environmental concentration distributions, the related levels of contaminants in different sources, and the fraction of a chemical release that will be taken in by the entire human population (the intake dose) at the regional or local scale. When the spatial resolution of computations is low, variations in environmental characteristics usually tend to average out, and adoption of roughly selected representative or characteristic values allows the correct orders of magnitude of outputs to be depicted. Research is starting to cope with spatially explicit models of fate and transport with increasing resolution, and now a few models with a resolution ranging from a few tens of a km up to 1 km are available for calculations at the continental scale [24, 42]. However, the computational effort associated with this modeling strategy is generally quite high and limits routine applications when a large number of chemicals need to be evaluated.

A multimedia fate and exposure model called Modul’ERS [43, 44] developed by INERIS is used to estimate intakes from air inhalation and soil, tap water, marketed food products, as well as eating locally produced fruits and vegetables. Local foodstuff concentrations are estimated using atmospheric deposition of particulate pollutants, air (for POP) and soil concentrations. As mechanistic and dynamic models for plants required many input data that can be difficult to define (lack of data, difficulty for estimating the magnitude of variability and uncertainty of data and even anticipating the qualitative effect of variations in the input data on results), the contributions of gaseous air and soil concentrations to edible plant organs are estimated from bioconcentration factors, which are specific to the different categories of fruit and vegetables cultivated in domestic gardens and time averaged concentration during cultivation. Therefore, the inputs of the model for media concentration estimates are georeferenced environmental databases (with a direct reuse of the treated data incorporated into the GIS for tap water and marketed food products).

In the model used, attention was focused on the quality of the values used to define all inputs (exposure, environmental and chemical parameters). The available data were systematically analyzed. For most of the parameters, all the data collected, together with their contextual information, as well as the selection criteria used, are described in dedicated reports. Depending on the level of knowledge, the quantity and the relevance of the data available, the parameters are finally defined with a point value, a range of values or a probabilistic distribution. The multimedia exposure model provides an external exposure dose that could be integrated into a physiologically-based pharmacokinetic (PBPK) model as input data.

### Physiologically-based pharmacokinetic models

PBPK models are a specific class of biokinetic models based on the physiology and the anatomy of the individuals. They can predict the kinetics and metabolism of substances in the body. These models provide realistic descriptions of xenobiotics’ absorption, distribution, metabolism, and excretion processes. They describe the body as a set of compartments corresponding to specific organs or tissues (e.g., adipose, bone, brain, gut, heart, kidney, liver, lung, muscle, skin, spleen, etc.). Between compartments, the transport of substances is dictated by various physiological flows (blood, bile, pulmonary ventilation, etc.) or by diffusion [25, 45]. The model structure can be described by a set of differential equations, with parameters representing blood flow rates, organ volumes etc., for which information is available in the published scientific literature or may be obtained in vitro [46]. Numerical integration of that differential system computes the quantity and concentration of the drug considered in each compartment, as a function of time and the exposure dose. Thus, those models offer a quantitative mechanistic framework to understand and simulate the time-course of the concentration of a substance in various organs and body fluids [47]. A stochastic whole-body physiologically-based pharmacokinetic model over the human lifespan has been developed by INERIS [47] and integrated in the EHI context to predict the internal concentration such as concentrations in blood but also in other tissues or biological matrices (urine) from multi-route exposure (inhalation, ingestion, dermal exposure). Those models are used to link exposure with biomarker data [26, 48] and have proven to be successful in integrating and evaluating the influence of age or gender-dependent changes with respect to the pharmacokinetics of xenobiotics throughout the lifetime [49].

Each model represents a different component of the emission-environmental quality-exposure-internal dose and effects continuum. This framework was thus designed to allow internal exposure assessments for different human populations (general population, pregnant women, children at different ages, socio-economic status, etc.) integrating exposure through multiple pathways. Integrated evaluations over the full chain were tested on a case study presented in this issue. These models can operate in different spatial-temporal scales, which poses a challenge when coupling them in a coherent framework and can result in structural uncertainty and a deep time calculation problem.

## Key illustrative case study

One of the objectives of the integrated approach was to demonstrate the feasibility of building complex realistic exposure scenarios satisfying the needs of stakeholders and the accuracy of the modelling predictions at a fine spatial-temporal resolution. This case study can be seen as a reference case that provides a specific application of the proposed framework and how the result could be used to identify an overexposed population.

To illustrate the approach, contamination of the general population is studied for a mixture of pyrethroids (cypermethrin [50] and deltamethrin) in the Picardy region (Northern France). A cypermethrin and deltamethrin exposure assessment was carried out in 2013 over the Picardy region in northern France. It is a moderately densely populated region, with an area of 19,399 km^2^ and almost 2 million inhabitants - 3% of France’s total population. Picardy is a region of field crops and one of the major consumers of pesticides. According to the French National Bank of Plant Protection Products Sales by Authorized Distributors (BNV-D), about 12 tons of cypermethrin and 6 tons of deltamethrin have been sold in the region and spread over 1.3 million hectares of agricultural land during this year, being used for cereal and vegetable crops, orchards and vineyards [51].

Exposure to pesticides are characterized by a multiplicity of exposure routes (food, water, soil, air) related to their presence in all environmental media. For a fine characterization of the environmental exposures, the first prerequisite resides in the capacity to gather a dataset, within the same analysis system, that combines population behavior and the local contamination of the environmental media at fine resolutions and over large territories. Modeling the fate and transport of pyrethroids between environmental components, exposure media and the population required the integration of databases (Table S1) allowing the characterization of pollutant sources in 2013 in Picardy, such as agricultural spreading the meteorological parameters [52] and the environmental concentrations of substances in water and food products [53–55].

**Fig. 2:**
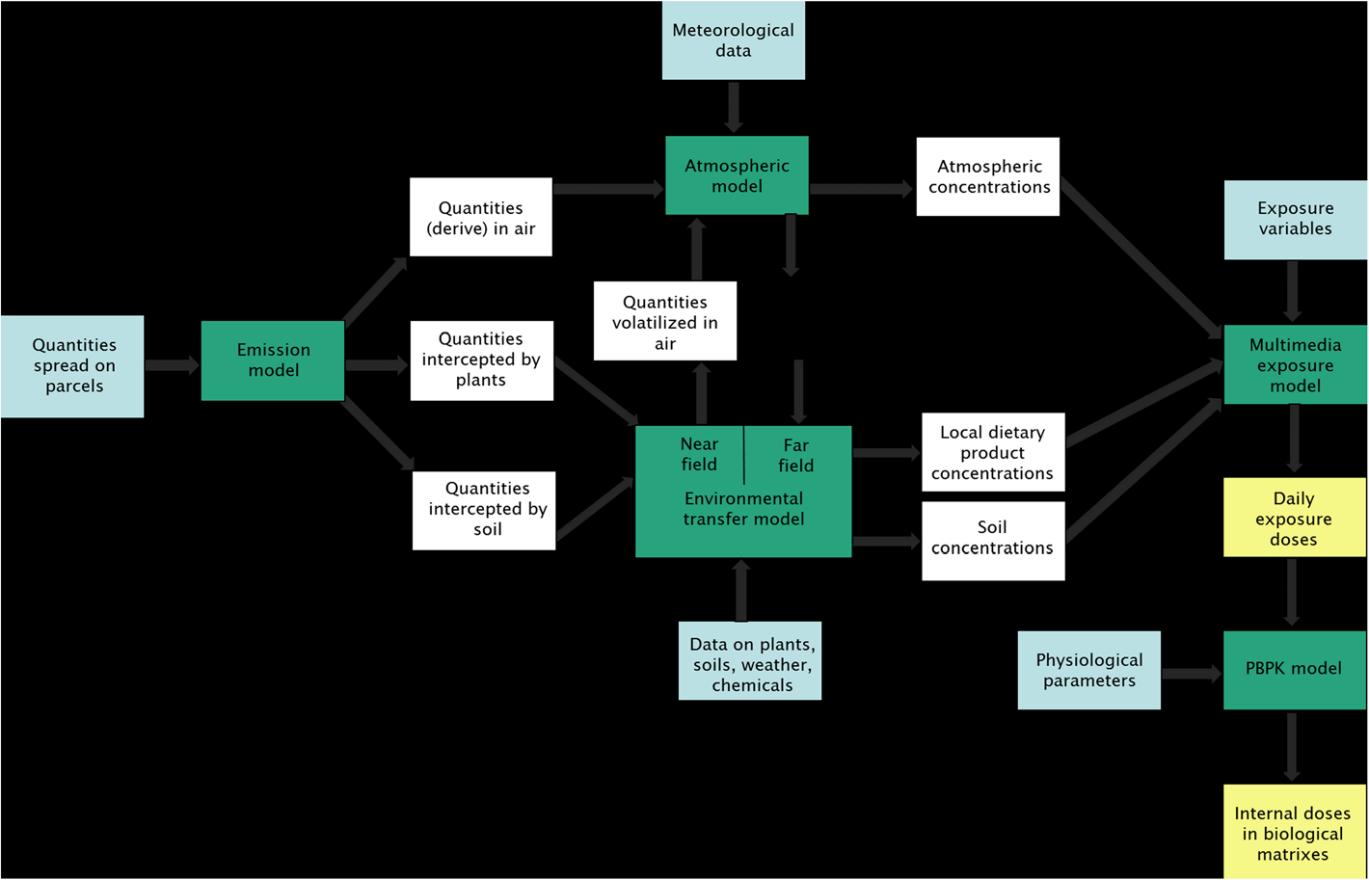
Conceptual scheme of the modeling approach used in this study. Environmental data (blue) are integrated into models (green) which characterize the transfers of pesticide from the source to contamination of the target populations. The output data generated by these models (white) are themselves integrated as input data for the following model. The external and internal exposure doses (yellow) are estimated at the end of the modeling chain

An approach integrating and coupling models with environmental data has been developed [56] and applied to this study (Fig. 2).

The models have been adapted to allow the integration of the output data from an upstream model as input data for the downstream model. One model assessed ambient air concentrations and deposits (far-field) by considering emission conditions either in particulate or gaseous form and meteorological data (near-field). Then, multimedia exposure models were used to estimate pollutant transfers between each environmental component (water, air, soil) to predict the exposure of pregnant women resulting from the integration of all media concentrations [43, 57]. In addition to this local exposure, the contribution of other exposure sources, e.g. non-local food products, were modelled to be added to total external exposure.

### Input data

#### Agricultural data

Annual quantities applied over agricultural parcels in Picardy in 2013 were estimated with data from the BNV-D [51]. Product sales extracted from this database were spatially distributed under a methodology built by the French National Institute for Agriculture, Food, and Environment (INRAE) according to the land use (crop type) and the postal code of the purchaser at field crop resolution. Based on data on the agricultural spreading times provided by the departmental agricultural chambers, the quantities applied were simulated at a tri-hour step.

#### Outdoor air

Parameters including wind, temperature, precipitation, humidity and cloudiness available at a 3-h frequency were extracted from the meteorological stations of Dieppe, Lille, Caen, Rouen and Orly, these framing the target region and describing a representative climate of northern France. Most notably for the atmospheric dispersion statistical model, the database of the Synop Essential network of surface stations of the World Meteorological Organization (WMO) was used [52].

#### Non-local food

Concentrations of cypermethrin and deltamethrin in non-local, i.e. commercial, food products came from the French Total Diet Study (EAT2) and European Food Safety Authority’s measurement compilations [53, 54]. Quantification frequencies of cypermethrin and deltamethrin in commercial food products were very low (both 0% for EAT2 study while 5.3 and 1.1% for the EFSA study respectively). Thus, two scenarios were determined to frame exposure to commercial products. The lower bound scenario (LB) is a minimalist scenario for which the undetected values are considered equal to 0 and the values detected but not quantified are estimated equal to the limit of detection. The upper bound scenario (UB) is a maximalist scenario for which the undetected values are considered equal to the limit of detection and the values detected but not quantified are estimated as equal to the limit of quantification. The values used in the upper bound scenario correspond to the maximum substance concentrations measured in commercial food products.

### Fate and transport modeling approach

#### Air concentrations and atmospheric deposits

Based on data from the BNV-D, departmental agricultural chambers and the simulations of the quantities applied at a tri-hour step, the distributions on soil, plant and air (drift) in the first minutes after spreading were estimated from Pest-LCI 2.0 (USEtox™) [58] combined with dynamiCROP [57] for each crop in the Picardy region concerned with pyrethroid use. Air drift, as well as emission fluxes from soil and plant volatilization, then fed the atmospheric model.

The large number of parcels, their heterogeneous boundaries and the strong temporal variability of emissions have directed the modeling approach towards a machine learning approach and the development of a statistical metamodel. A database resulting from ADMS (Numtech, version 5.2) simulations was thus constituted based on emissions of one basic parcel and meteorological parameters observed at surface stations. This database was then used to learn the statistical model. Once the model was calibrated and validated (Fig. S1), it was applied to all parcels and provided atmospheric deposits and concentrations of cypermethrin and deltamethrin either in the gaseous or particulate form at a three-hourly interval. The form of the substance was assumed not to evolve after emission and was considered to be a passive tracker.

#### Multimedia exposure model

Multimedia exposure modeling follows a mass balance approach and is based on the resolution of differential equations with first-rate kinetics. The modeling was performed with two multimedia models, dynamiCROP for the assessment of environmental transfers, and Modul’ERS to estimate cypermethrin and deltamethrin daily exposure doses from environmental components (water, air, soil and food).

Contamination of local food products cultivated in vegetable gardens was estimated from atmospheric deposits on plants and root uptake from soil. Four crops corresponding to the main food products self-consumed were studied: apple, lettuce, potato and tomato. For each crop, one plant model was used to integrate transfer specificities (leaf, fruit, root, tuber). Transfers between plant, air and soil were estimated from the dynamiCROP model which was conveyed to the Python language to reduce the large computing times generated by Excel and MATLAB®. Pyrethroid concentrations were estimated at harvest time. Lettuce being a crop harvested all year round, all concentrations estimated each week of the year were weighted according to the probability of harvesting based on the evolution of the leaf area index (LAI).

One model was provided for each crop studied (apple, lettuce, potato, tomato) according to their specificities. In each model, the calculation method of some parameters was redefined from the initial model. The parameters concerned were related to the biomasses (mass, volume, area) of plant compartments (fruit, leaf, root, stem), transfer coefficients between plant compartments and flow rates (xylem, phloem). The initial model calculated these parameters considering average biomass values over the year. The new calculation method considers biomass values that change over time.

A percentage of self-consumption was defined from INSEE data [59]. It corresponds to the difference between total food consumption per person and consumption of commercial products. Four scenarios were defined, based on the number of inhabitants per urban unit (1) a municipality with less than 2000 inhabitants (2) between 2000 and 10,000 (3) between 10,000 and 100,000 (4) with more than 100,000 (Fig. S2).

Consumption of commercial food products was estimated using concentration measurements from French and European studies and mean dietary beverage consumption from a French study. Aggregated multimedia exposure was then assessed from ingestion and inhalation pathways. The inhalation pathway was given as the sum of pyrethroid concentrations in their particulate and gaseous forms. The ingestion pathway was estimated considering weight, the quantities of food products ingested, water consumption, soil ingestion and a self-consumption factor characterizing the ingestion of local food products (Fig. S3).

#### Toxicokinetic modeling and internal exposure

To model the pharmacokinetic behavior of cypermethrin and deltamethrin as well as a common metabolite: 3-PBA, a PBPK/PD model was used that has been developed by Quindroit et al. [26]. The model structure includes 23 tissue compartments for the parent compounds and one urinary compartment for the metabolites. It gives estimations of organ and blood concentrations of the parent compounds as well as urinary excretion of 3-PBA. This model takes into account changes in physiology, metabolism and sensitivity to toxicity over life-stages from childhood to adulthood and multi-route exposure. It was parameterized from animal in vivo experiments, in vitro human cells and in silico estimates (QSAR models). Urinary excretion of 3-PBA was calibrated from experimental data on human volunteers.

#### Statistical analysis

Pyrethroid exposure was assessed over a weekly time period and a regular grid with a spatial resolution of 4 km^2^. The regular grid constituted a common spatial unit on which all the data were described.

In order to construct exposure maps from spatialized databases, several statistical methods were used to specifically address environmental, behavioral or populational databases to increase their representativeness regarding the objectives of exposure characterization. Data processing methods were adapted from the GIS-based modeling platform PLAINE. Statistical and geoprocessing methods interfaced in a GIS were primarily used to bypass the issues generated by data gaps and estimate exposure indicators in the areas of interest.

Since air inputs were the main environmental spatial determinant considered in the modeling, a geostatistical analysis was conducted on atmospheric concentrations and deposits to assess spatial autocorrelations. This analysis was conducted in order to (1) better define a relevant grid spatial resolution for reducing computation time and (2) investigate the possibility of estimating exposure at a specific point (i.e. the located address of a cohort participant to compare with a measured biomarker) using the initial grid calculation. This analysis consisted of studying the sample 2D-variogram and testing the spatial anisotropy.

### Environmental inequality determinant analysis

**Fig. 3:**
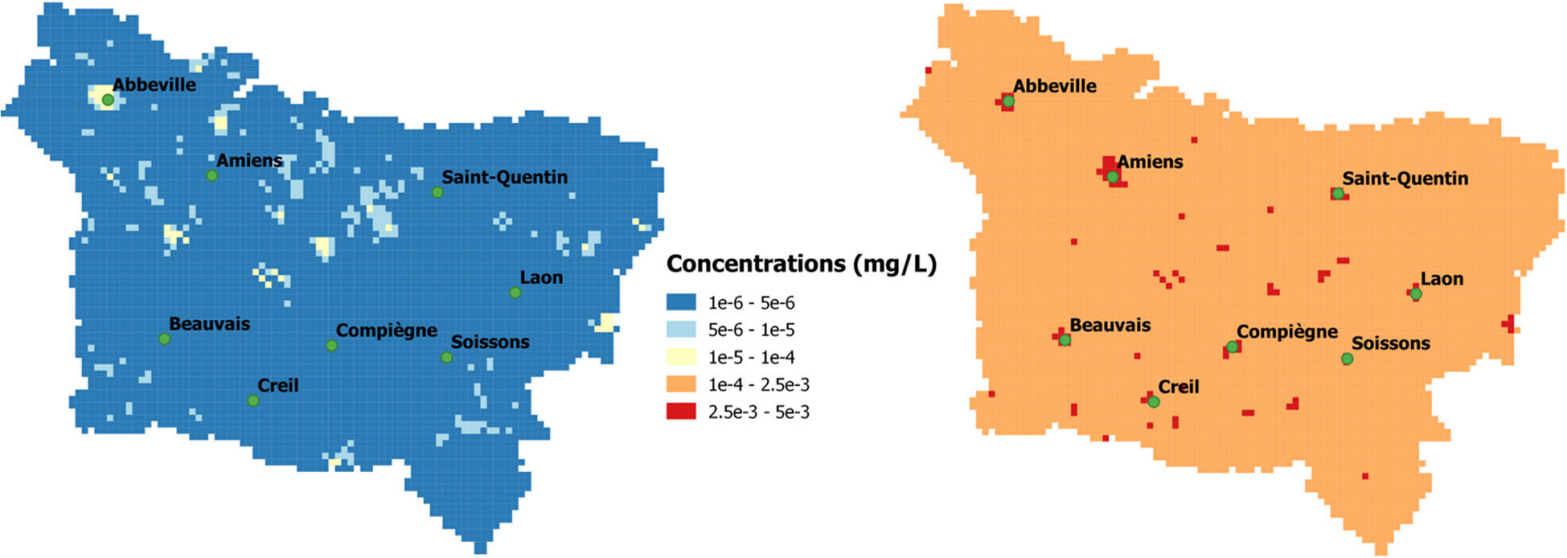
Mapping for urinary concentrations of 3-PBA in the general population. **a** Mean annual urinary concentrations (lower bound); **b**) Mean annual urinary concentrations (upper bound)

Annual mean 3-PBA urinary concentrations, resulting from the aggregation of inhalation and ingestion pathways, are comprised between 1.4 × 10^− 6^ and 8.6 × 10^− 5^ mg/L in the lower bound scenario. With the upper bound scenario, the range of 3-PBA urinary concentrations varies by 5% for the annual mean and fall between 2.5 × 10^− 3^ and 2.6 × 10^− 3^ mg/L (Fig. 3).

**Fig. 4:**
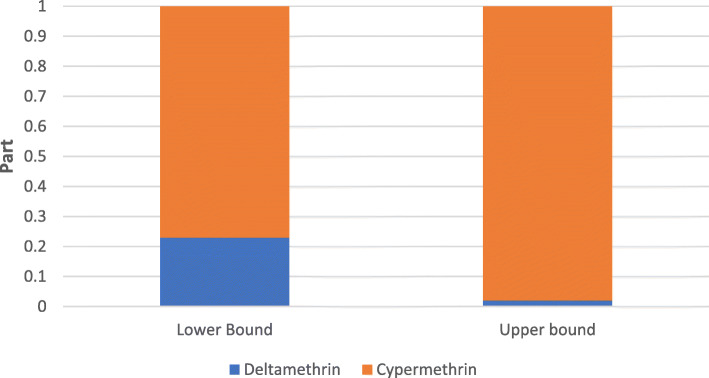
Mean contributions of pyrethroids to cumulated 3-PBA urinary concentrations for the lower bound and upper bound scenarios

The contributions of each parent compound to 3-PBA urinary concentrations vary depending on dietary assumptions. Cypermethrin counts for 76% of total 3-PBA urinary concentrations in the lower bound scenario and reaches 98% in the upper bound scenario (Fig. 4). The significant contribution made by cypermethrin compared to deltamethrin can be explained by higher concentrations in the environmental components and commercial food products.

## Conclusion

The exposome concept has been proposed as an emergent exposure science paradigm for conceptualizing the cumulative effects of environmental exposures across an entire human life. The need for a risk manager to identify a population at-risk in the context of substantial data deficiencies, which hinder the evaluation of cumulative health risks, means there is an operational decline in the concept at the territorial scale in the context of EHI characterization. The characterization of the territorialized exposome implies the development of dynamic, multidimensional, longitudinal approaches, and information systems that require the adoption of transdisciplinary methods of data analysis. For example, integrated approaches bring together all the information necessary for assessing the source-to-human-dose continuum using GIS, multimedia exposure and the toxicokinetic model.

This framework could be used for many purposes, such as:

- mapping EHI;

- identifying vulnerable populations and determining exposure to manage and plan remedial actions;

- highlighting hotspots with significantly elevated exposure indicator values to define environmental monitoring campaigns;

- assessing spatial relationships between health, socioeconomic and environment to identify factors that influence the variability of disease patterns or environmental injustice.

## Supplementary Information

**Additional file 1.**

## Data Availability

The datasets used and/or analyzed during the current study are available from the corresponding author upon reasonable request.
