# Supplementary material for: Characterizing environmental geographic inequalities using an integrated exposure assessment
Source: Environ Health. 2021 May 12;20:58. doi: 10.1186/s12940-021-00736-9 (PMC8117491; doi:10.1186/s12940-021-00736-9)
Supplement: Supplementary file 1 — Additional file 1. [file 12940_2021_736_MOESM1_ESM.docx]

**Supplemental materials**

# Characterizing environmental inequities using an integrated exposure assessment and spatial approach

Julien CAUDEVILLE ^1,3^*, Corentin REGRAIN ^1,2,3^, Frederic TOGNET ^1^, Roseline BONNARD ^1^, Mohammed GUEDDA^2^, Celine BROCHOT^1^, Maxime BEAUCHAMP^1^, Laurent LETINOIS^1^, Laure MALHERBE^1^, Fabrice MARLIERE^1^, Francois LESTREMAU^1^, Karen CHARDON^3^, Veronique BACH^3^, Florence Anna ZEMAN^1^

* Correspondence: [caudevillej@yahoo.fr](mailto:julien.caudeville@ineris.fr)

^1^ Institut National de l'Environnement Industriel et des Risques (INERIS), Parc ALATA BP2, 60550 Verneuil en Halatte, France

^2^ LAMFA, UMR CNRS 7352, Université de Picardie Jules Verne, 33 rue Saint-Leu, 80039 Amiens, France

^3^ PériTox, UMR_I 01, CURS, Université de Picardie Jules Verne, Chemin du Thil, 80025 Amiens, France

**Table of contents**

**Figure S1 – Representation of the values predicted (by the statistical model against the ADMS simulation values on the test dataset.**

**Figure S2 – Location of urban zones in Picardy according to the typology of INSEE (>2000 inhabitants).**

**Figure S3 – Mapping daily exposure doses for the inhalation and ingestion pathways for the general population in Picardy.**

**Table S1 – Available data and use.**

**References**


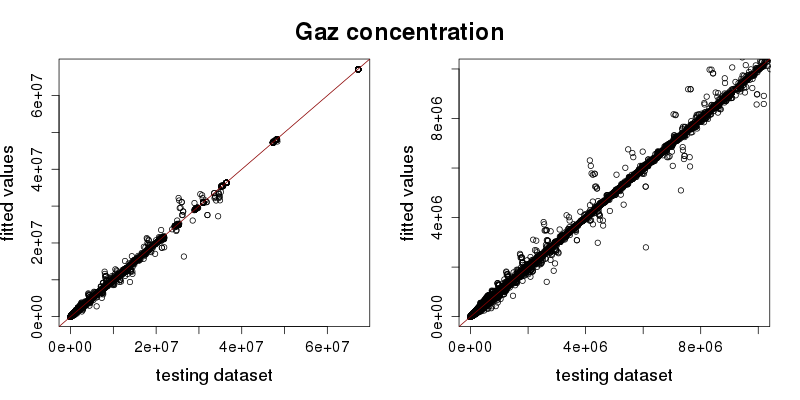

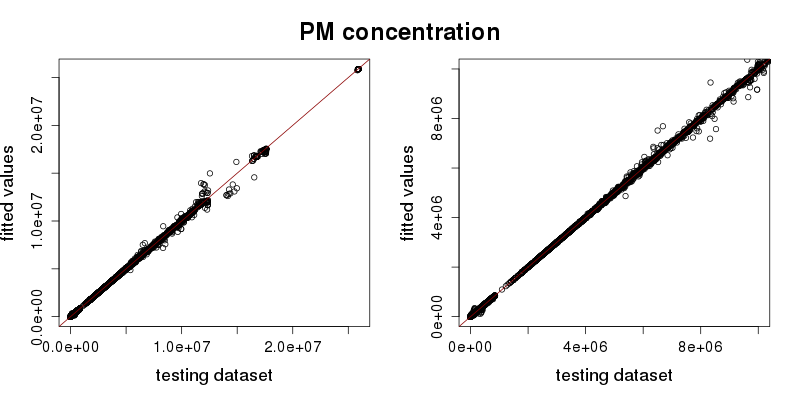

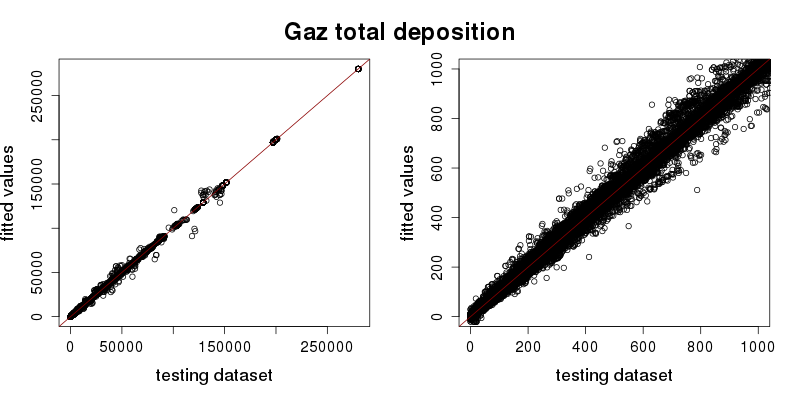

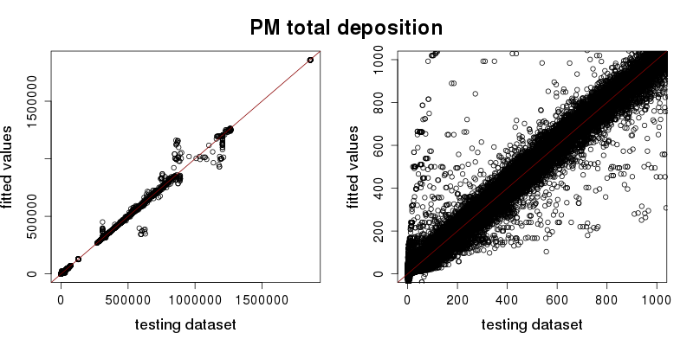


**Figure S1 : Representation of the values predicted (total deposits in µg.m-2.s-1 and concentrations in ng.m-1) by the statistical model against the ADMS simulation values on the test dataset and a focus on the low values for a gaseous species (left) and a particulate species (right).**

**Figure S2 – Location of urban zones in Picardy according to the typology of INSEE (>2000 inhabitants).**


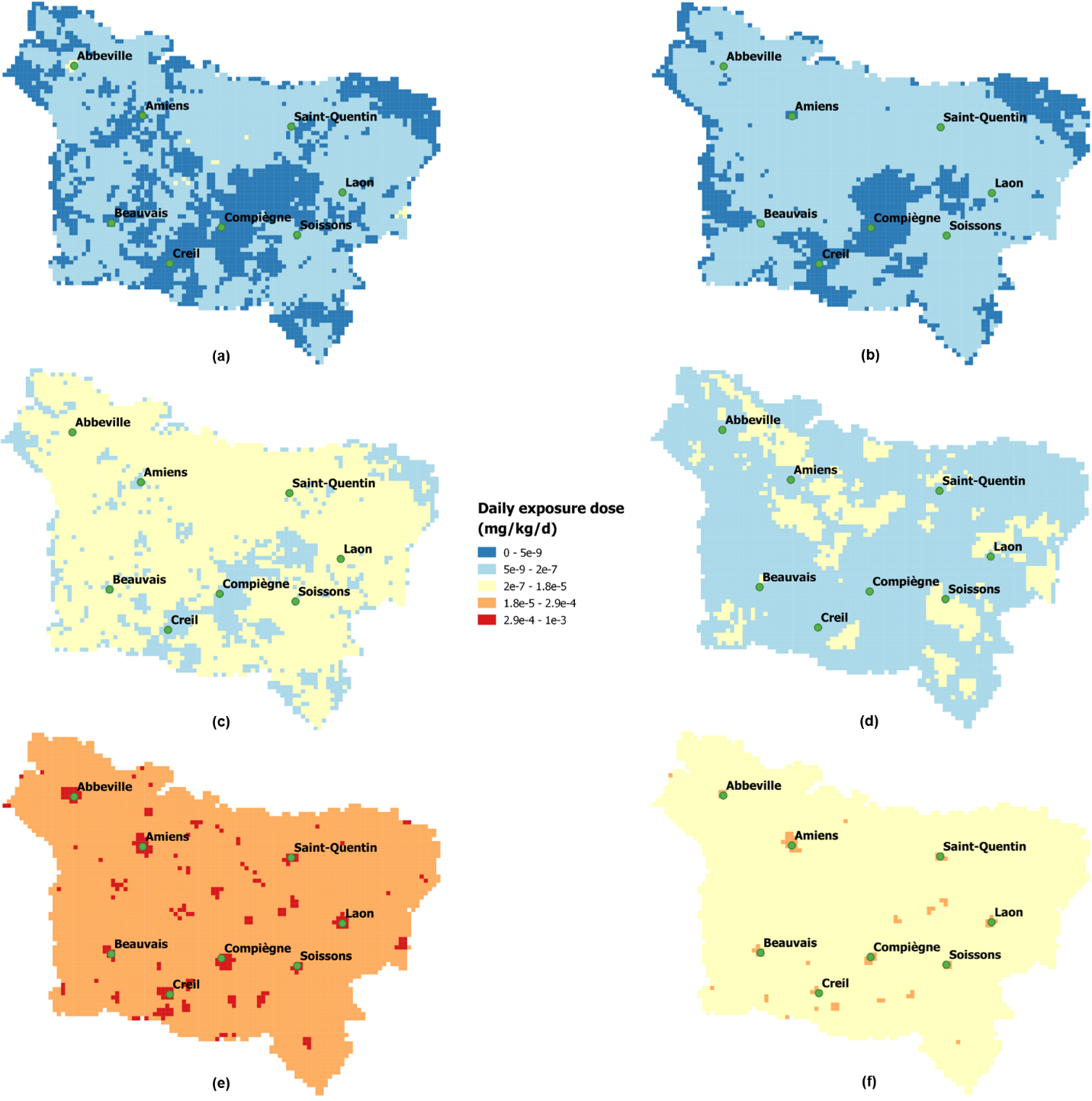


**Figure S3 : Mapping daily exposure doses for the inhalation and ingestion pathways for the general population in Picardy. A) Mean annual daily inhalation doses of cypermethrin; B) Mean daily inhalation doses of deltamethrin; C) Mean annual daily ingestion doses of cypermethrin (lower bound); D) Mean daily ingestion doses of deltamethrin (lower bound); E) Mean daily ingestion doses of cypermethrin (upper bound); F) Mean daily ingestion doses of deltamethrin (upper bound).**

**Table S1 – Available data and use.**

| Variable | Data source and use |
| --- | --- |
| Plant protection product sales | National Bank of Plant Protection Products Sales by Authorized Distributors (BNV-D, 2020). To be used to predict annual quantities applied. |
| Spreading times | Departmental agricultural chambers (Chambres d’Agriculture Hauts-de-France, 2020). To be used to predict annual quantities applied. |
| Wind Temperature Precipitations Humidity Cloudiness | Synop Essential WMO (Synop Essentielles OMM, 2020). To be used to predict atmospheric dispersion. |
| Concentrations in commercial food products | French Total Diet Study (Leblanc et al., 2011) and EFSA (EFSA, 2015). To be used to predict dietary ingestion exposure. |
| Concentrations in tap water | SISE-Eaux (Davezac et al., 2008). To be used to predict water consumption exposure. |

**References**

BNV-D (National Bank of Plant Protection Products Sales by Authorized Distributors). 2020. http://www.data.eaufrance.fr/jdd/bd45f801-45f7-4f8c-b128-a1af3ea2aa3e. Accessed 13 march 2021.

Chambres d’Agriculture Hauts-de-France (Hauts-de-France Agricultural Chambers). Chambre regional d’agriculture, Lille. 2020. https://hautsdefrance.chambres-agriculture.fr/voschambres/. Accessed 13 march 2021.

Davezac H, Grandguillot G, Robin A, Saoult C. L’eau potable en France 2005–2006. French Ministry for Health, Youth and Sports: Paris. 2008.

EFSA. The 2013 European Union report on pesticide residues in food. EFSA Journal. 2015;13 (3):4038; doi:10.2903/j.efsa.2015.4038.

Leblanc JC, coordination, Sirot V, coordination, et al. Étude de l’alimentation totale française 2 (EAT 2) - Tome 2. Agence nationale de sécurité sanitaire de l’alimentation, de l’environnement et du travail: Maisons-Alfort. 2011. French.

Synop Essentielles OMM. Synop Essential WMO Database. Météo-France, Saint-Mandé. 2020.https://donneespubliques.meteofrance.fr/?fond=produit&id_produit=90&id_rubrique=32. Accessed 13 March 2021.
